# The history of *Wolbachia* and heartworm

**DOI:** 10.1186/s13071-026-07397-y

**Published:** 2026-08-20

**Authors:** A. Vismarra, M. Fozzer, J. W. McCall, M. Genchi, L. Venco, R. Morchón García, C. Bazzocchi, C. Genchi, L. Kramer, C. Bandi

**Affiliations:** 1 Department of Veterinary Medicine Sciences, University of Parma, Strada del Taglio, 10, 43126 Parma, Italy; 2 TRS Labs Incorporated, Athens, GA USA; 3 Department of Veterinary Sciences, University of Turin, Turin, Italy; 4 Zoonotic Diseases and One Health Group, Faculty of Pharmacy, Biomedical Research Institute of Salamanca-Research Centre for Tropical Diseases University of Salamanca (IBSAL-CIETUS), University of Salamanca, 37007 Salamanca, Spain; 5 Department of Veterinary Medicine and Animal Science, University of Milan, Lodi, Italy; 6 Department of Biosciences, University of Milan, 20133 Milan, Italy

**Keywords:** *Wolbachia*, *Dirofilaria immitis*, History, Endosymbiosis

## Abstract

*Wolbachia* is the most widespread endosymbiont in animals, and its discovery in *Dirofilaria immitis* significantly advanced our understanding of heartworm disease (HWD). In 1995, Sironi and colleagues from the universities of Milan and Pavia, Italy, published a study titled “Molecular evidence for a close relative of the arthropod endosymbiont *Wolbachia* in a filarial worm” (Mol Biochem Parasitol. 74:223–7, 1995). In the 1970s, intracellular bacteria were observed in filarial nematodes by electron microscopy, but no one understood what they were or why they were there. There had been previous reports on the anti-filarial effects of tetracycline, but no connection with these bacteria had been made. Many years later, these bacteria were identified as belonging to the genus *Wolbachia*, marking a new chapter in filarial research. As soon as *Wolbachia* had been described in heartworms, there was a flurry of excitement in the scientific community. By 1999, *Wolbachia* had been described in a further nine species of animal and human filarial nematodes. Subsequent studies strongly suggested that *Wolbachia* are obligatory mutualist symbionts of *D. immitis*, and related research then focused on targeting *Wolbachia* in the treatment of HWD. Studies on the effects of doxycycline treatment in *D. immitis*-infected dogs reported that bacterial depletion in adult worms, elimination of microfilariae, and adult worm death were more effective when antibiotics were combined with macrocyclic lactones, and that use of this combination also avoided the deleterious post-adulticide effects of treatment with melarsomine. *Dirofilaria immitis*-infected dogs develop an immune response to *Wolbachia*, which likely contributes to the immunopathology of HWD. Insights into how *D. immitis* and *Wolbachia* interact were gained in 2012, when genome sequencing of these organisms was completed.

**Graphical Abstract:**

**Figure:**
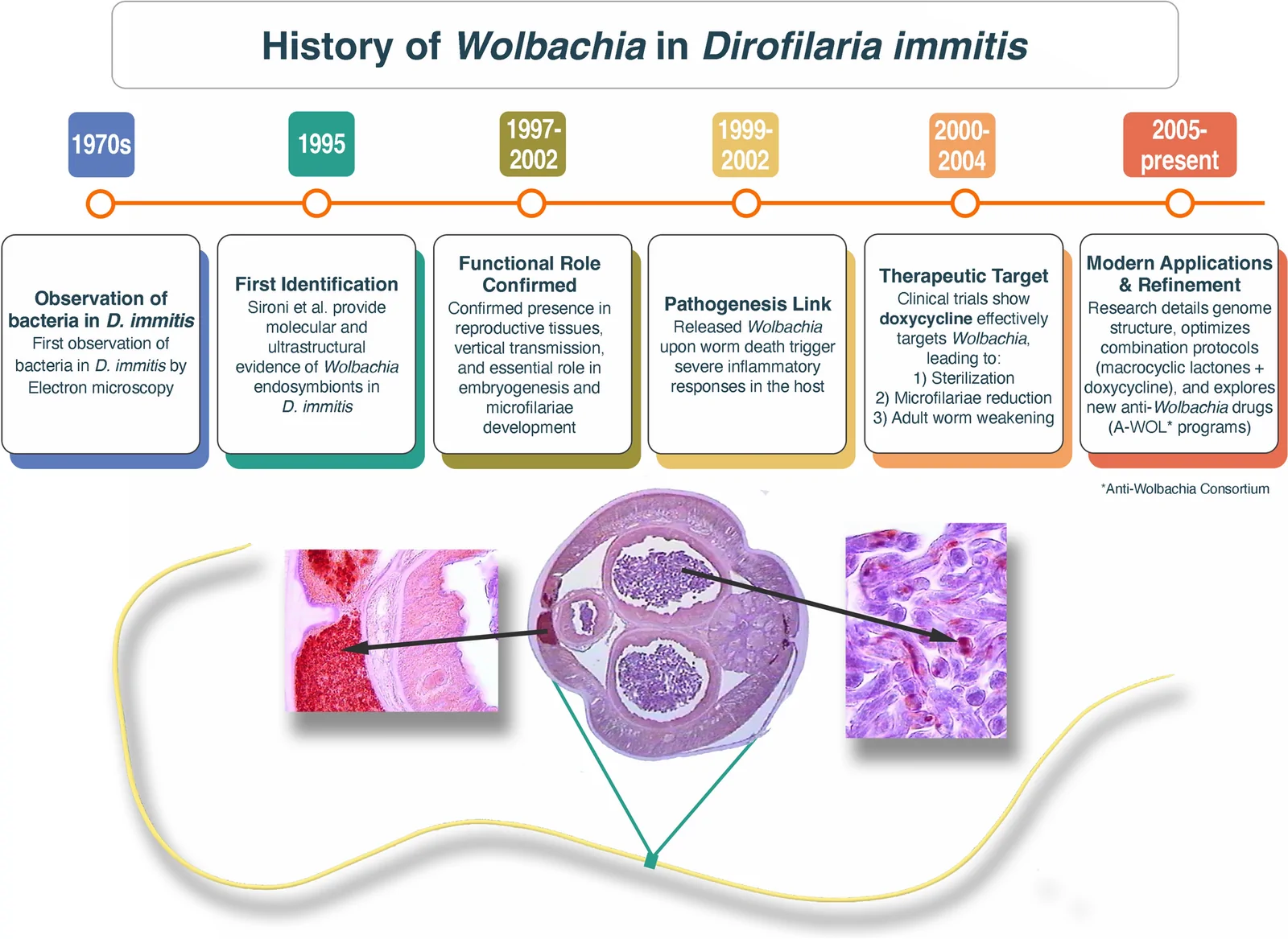

## Background

Endosymbiosis is the term used to describe the coexistence of two organisms where one lives inside the other. The word ‘endosymbiont’ is derived from two Greek root words: ‘endo’, meaning within, and ‘symbiont’, meaning living together [1]. *Wolbachia*, which were discovered in arthropods nearly 100 years ago [2], are the most widespread bacterial endosymbionts in animals. They are maternally inherited intracellular bacteria and are present in approximately 50% of all arthropod species [2]. In arthropods, *Wolbachia* act as reproductive parasites, causing a wide range of effects. For example, cytoplasmic incompatibility occurs when eggs fertilized by sperm from an uninfected male, or from a male infected with a different *Wolbachia* strain, fail to develop. Certain strains of *Wolbachia* kill male embryos (male killing), which ensures that only females survive and thus allows for the transmission of the bacteria to their offspring. Another effect of *Wolbachia* is parthenogenesis, where unfertilized eggs develop into female offspring, allowing the bacteria to spread in populations that are otherwise unable to reproduce sexually. Finally, *Wolbachia* can convert genetic male offspring into females through the phenomenon of feminisation, thereby promoting the spread of the infection.

*Wolbachia* was first discovered in a filarial nematode, *Dirofilaria immitis*, the causative agent of canine and feline heartworm disease (HWD), in 1995 [3]. This important discovery sparked a new wave of research. In this review, we highlight several key milestones in the history of research on *Wolbachia* and *Dirofilaria immitis*.

## *Wolbachia* and heartworm: how it all began

In 1995, colleagues at the universities of Milan and Pavia, Italy, published a study titled “Molecular evidence for a close relative of the arthropod endosymbiont *Wolbachia* in a filarial worm” [3]. However, the story of this discovery goes back much further. In 1971, Kozek [4], using electron microscopy, observed intracellular bacteria in the microfilariae of *D. immitis*, and referred to them as part of the *Innenkorper* (central body) of the larvae. McLaren et al. [5] observed ‘unusual bodies’ in the hypodermal tissues of larval *D. immitis* and *Brugia pahangi*, with ultrastructural evidence indicating that they were likely Gram-negative microorganisms. However, at that point, the role of these bacteria in filarial biology remained unknown. A study was also published on the anti-filarial effects of tetracycline, although at that point no one took into consideration the bacteria, believing instead that the antibiotic had a direct effect on the worms [6].

In the early 1990 s, Claudio Bandi (one of the authors of [3]) collaborated with Luciano Sacchi, based at the University of Pavia, to study endosymbionts in cockroaches at the University of Milan, where Bandi was a PhD student. Bandi had read the earlier reports of intracellular bacteria that had been observed in several filarial species. He was particularly intrigued by the fact that these bacteria were found in the larval stages of the nematodes and in the reproductive tract, which was highly suggestive of transovarial transmission, an important feature of arthropod endosymbionts [2]. Stimulated by scientific curiosity, and based on his expert knowledge of endosymbiosis, Bandi hypothesized that filarial nematodes could also harbour endosymbiotic bacteria. To search for possible intracellular bacteria, electron microscopy was performed on fresh heartworms, which were supplied by C. Genchi and L. Venco who surgically removed them from naturally infected dogs. Bacteria were found to be abundant in the ovaries, embryos, and body wall of the female nematodes [3]. PCR amplification and direct sequencing of 16S rDNA from the ovaries and uterus of female *D. immitis*, where the bacteria were most abundant, revealed nucleotide divergence of approximately 4–7% relative to arthropod *Wolbachia* sequences, indicating that the intracellular bacteria were phylogenetically closely related to this genus [3]. Thus, through the identification of the bacteria residing in adult heartworms [3], a new avenue of filarial research opened up.

## A flurry of excitement

As soon as Sironi and colleagues had described *Wolbachia* in heartworms [3], there was a flurry of excitement in the scientific community. In humans, the filarial nematodes *Brugia malayi, Wuchereria bancrofti*, and *Onchocerca volvulus* cause severe, debilitating diseases, and the search for effective treatments and control strategies for these parasites had been a major research goal for many years [7]. The discovery of *Wolbachia* in heartworm led scientists around the world to question if it was present in other filarial nematodes, and by the end of 1999, it had been described in a further nine species of animal and human filarial nematodes (Table 1). Early results indicated 100% prevalence of *Wolbachia* in filarial species that were positive at fixation, and there was also strong evidence for the co-evolution of filarial nematodes and their *Wolbachia* symbionts (matching phylogenies) [8, 9]. However, later studies indicated that the presence of *Wolbachia* in some filarial species is patchy, and that the phylogenies of the host nematode and *Wolbachia* do not always align [10]. Despite these findings, the phylogenies of filarial nematodes and *Wolbachia* are largely congruent across substantial portions of their evolutionary trees, with the major filarial species of clinical importance in humans and dogs, including *D. immitis*, showing 100% prevalence of the symbiont.

**Table 1:** Species of major filarial nematodes known to harbour *Wolbachia*

Species	Host(s)	Disease	References
*Dirofilaria immitis*	Canids (felids)	Heartworm disease	[3]
*Brugia pahangi*	Felids and canids	Lymphatic filariasis	[8]
*Brugia malayi*	Humans	Lymphatic filariasis	[8]
*Wuchereria bancrofti*	Humans	Lymphatic filariasis	[8]
*Onchocerca volvulus*	Humans	Onchocerciasis (river blindness)	[10]
*Mansonella perstans*	Humans	Perstans filariasis	[45]
*Onchocerca ochengi*	Bovines	Nodular skin disease	[10]
*Onchocerca gibsoni*	Bovines	Nodular skin disease	[9]
*Onchocerca gutturosa*	Bovines	Nodular skin disease	[10]
*Dirofilaria repens*	Canids (felids), humans	Subcutaneous dirofilariasis	[31]
*Litomosoides sigmodontis*	Rodents	Peritoneal filariasis	[9]

Naturally, researchers began to question whether *Wolbachia* could serve as a target for the treatment of filarial infection. In addition, several authors reported that the anti-filarial effects of tetracycline observed in the early 1990 s had been due to the depletion/elimination of *Wolbachia*.

Bandi et al. [11] showed that tetracycline treatment effectively blocked embryo development in *B. pahangi* and *D. immitis*. Tetracycline therapy also induced infertility in the filarial nematode *Litomosoides sigmodontis* [12]. Treatment with oxytetracycline of cattle infected with *Onchocerca ochengi* led to the death of the adult worms, the resolution of skin nodules, and was associated with a nearly complete loss of intracellular *Wolbachia* [13]. In vitro and in vivo trials with several antibiotics revealed activity against *Onchocerca gutturosa, Onchocerca lienalis*, and *B. pahangi* [14].

These studies led to the founding, in 2007, of the Anti-*Wolbachia* Consortium—which is funded by the Gates Foundation—with the aim of finding new drugs targeting *Wolbachia* for the treatment of human filarial diseases [15].

## *Wolbachia*,* D. immitis*, and HWD

A detailed description of the distribution of *Wolbachia* in *D. immitis* was reported in 2003 [16]. The bacteria are present throughout the nematode’s life cycle, including in the adult worms, and are predominantly found in the hypodermal cells of the lateral cords. In the females, they are also present in the ovaries, oocytes, and developing embryonic stages within the uteri. However, they have not been observed in the male reproductive system, suggesting that they are maternally transmitted via the egg’s cytoplasm.

In 2005, it was shown that *Wolbachia* is released following the worm’s death (natural or adulticide induced) and through microfilarial turnover in dogs infected with heartworm [17]. *Wolbachia* surface protein, which is an antigen, has been observed in various tissues from dogs with natural HWD, and in particular in tissues of the lungs and kidneys. Even though *Wolbachia* do not infect mammalian cells, several studies showed that *Wolbachia*-derived molecules invoke classic inflammatory responses upon release in mammals, including activation of the pro-inflammatory mediators Toll-like receptors 2/6, neutrophil recruitment, NETosis, and an increase in specific immunoglobulins [18–25]. This strongly suggested that *Wolbachia* play a role in the inflammatory pathology associated with HWD, especially after the treatment of infected individuals with adulticides. This led to further studies designed to evaluate the benefits of removing *Wolbachia* by adding antibiotics to adulticide treatment protocols for heartworm (see below).

The decade from 1999 to 2008 witnessed significant advances in our understanding of the role of *Wolbachia* in canine HWD, and its potential as a therapeutic target. In 2008, a study on experimentally infected dogs showed that bacterial depletion in adult worms, the elimination of microfilariae, and adult worm death were all more effective when doxycycline was combined with macrocyclic lactones (MLs) [26], and that this combination also reduced the potentially severe post-adulticide effects of melarsomine [27]. This reduction of post-adulticide effects is likely due to the depletion of *Wolbachia*-derived pro-inflammatory molecules. Furthermore, a reduction in the number of intrauterine embryos and microfilariae may lower the risk of allergic-type inflammatory reactions, including mast cell degranulation [28].

Based on these studies, international guidelines were revised to recommend that melarsomine be administered after a month of doxycycline and monthly preventive doses of MLs, with the three-injection melarsomine protocol starting 2 months later [29, 30]. A retrospective study of dogs with HWD reported that adding doxycycline to canine heartworm treatment protocols reduces post-treatment complications and mortality in naturally infected clinical cases [31].

## The combination of doxycycline and a ML as an alternative adulticide

Since 2010, numerous field studies have been undertaken to evaluate whether a combination of doxycycline and an ML could serve as an alternative to melarsomine for the treatment of heartworm infection in dogs. Melarsomine is not available in many countries, may be contraindicated in some cases, and may be too costly in certain contexts [32]. Most of these studies evaluated the combination of monthly topical moxidectin and 30 days of doxycycline at 10 mg/kg, administered once daily or twice daily, with moxidectin administered monthly until no antigen was detected. Under this treatment, most dogs will be antigen-negative by 12–18 months [33]. Based on the results of these field trials, the international guidelines were revised and now recommend that, in selected cases (i.e. when melarsomine is unavailable, severe disease is not present, and there is the possibility of restricting exercise for the entire treatment period), the use of a combination of doxycycline and ML may be considered [29, 30]. This alternative protocol appears to be well tolerated. However, under this protocol, heartworm-induced pneumonitis and pulmonary thromboembolism may present at unpredictable times, and close monitoring of patients is fundamental to its use [34].

## Research prospects

The discovery of *Wolbachia* in *D. immitis* was groundbreaking and led to the development of novel treatment strategies for filarial diseases. In 2019, the World Health Organization recommended the use of doxycycline to achieve the elimination of onchocerciasis in the Americas [35]. Research is now aimed at identifying new anti-*Wolbachia* molecules [36]. Based on knowledge gained from the complete genome sequencing of *D. immitis* and its *Wolbachia* [37], we know that these bacteria are unable to synthesize several vitamins and cofactors (coenzyme A, biotin, folate), making them metabolically dependent on their *D. immitis* host. On the other hand, known effects of the elimination of *Wolbachia* from *D. immitis* (arrested larval development and loss of fertility and vitality of adult filarial worms) are likely due to a lack of pathways for the synthesis of heme, purine, and pyrimidine in *Dirofilaria*, which are, however, present in *Wolbachia*. Thus, targeting one or more of these metabolic pathways may be key to developing new strategies against heartworms.

Research is presently aimed at identifying alternatives to doxycycline, due to the extensive period of time needed for bacterial depletion, the risk of selection for antimicrobial resistance, and the potential gastrointestinal side effects associated with this drug. Research carried out by the Anti-*Wolbachia* Consortium has identified several fast-acting, short-course anti-*Wolbachia* drugs, which are in development for the treatment and potential prevention of filarial disease [38]. One of these, AWZ1066S, has been shown to effectively deplete *Wolbachia* in *D. immitis* larvae in an immunodeficient mouse model of HWD [39].

Although *Wolbachia* has been identified in *Dirofilaria repens* [8, 40], we do not know its role in the pathogenesis of subcutaneous dirofilariasis, nor whether doxycycline/ML combinations are adulticidal. Several case reports of human and canine subcutaneous dirofilariasis have shown that doxycycline alone or in combination with MLs can clear circulating microfilariae for extended periods of time [41–43]. However, there is currently no diagnostic test available with which to confirm adulticide activity. We also do not know if these combinations are effective for the treatment of feline HWD, although the current American Heartworm Society Feline Guidelines [44] do state that, with the owner’s consent, doxycycline plus ML can be considered for use as a treatment of this disease.

## Conclusions

There is still much to do. However, the groundbreaking studies cited here confirm that targeting *Wolbachia* is a winning strategy for the treatment of HWD, and that it has led to the improved health and well-being of canine patients worldwide.

## Data Availability

Not applicable.

## Abbreviations

*D. immitis*: *Dirofilaria immitis *
HWD: Heartworm Disease
ML: Macrocyclic lactone
MLs: Macrocyclic Lactones
A-WOL: Anti-*Wolbachia *

## References

[1] Wernegreen JJ. Endosymbiosis. Curr Biol. 2012;22:R555–61. 10.1016/j.cub.2012.06.010. 22835786 10.1016/j.cub.2012.06.010

[2] Kaur R, Shropshire JD, Cross KL, Leigh B, Mansueto AJ, Stewart V, et al. Living in the endosymbiotic world of *Wolbachia:* a centennial review. Cell Host Microbe. 2021;29:879–93. 10.1016/j.chom.2021.03.006. 33945798 10.1016/j.chom.2021.03.006 PMC8192442

[3] Sironi M, Bandi C, Sacchi L, Di Sacco B, Damiani G, Genchi C. Molecular evidence for a close relative of the arthropod endosymbiont *Wolbachia* in a filarial worm. Mol Biochem Parasitol. 1995;74:223–7. 10.1016/0166-6851(95)02494-8. 8719164 10.1016/0166-6851(95)02494-8

[4] Kozek WJ. Ultrastructure of the microfilaria of *Dirofilaria immitis*. J Parasitol. 1971;57:1052–67. 4333387

[5] McLaren DJ, Worms MJ, Laurence BR, Simpson MG. Micro-organisms in filarial larvae (Nematoda). Trans R Soc Trop Med Hyg. 1975;69:509–14. 10.1016/0035-9203(75)90110-8. 1228988 10.1016/0035-9203(75)90110-8

[6] Bosshardt SC, McCall JW, Coleman SU, Jones KL, Petit TA, Klei TR. Prophylactic activity of tetracycline against *Brugia pahangi* infection in jirds (*Meriones unguiculatus*). J Parasitol. 1993;79:775–7. 8410553

[7] Risch F, Kazakov A, Specht S, Pfarr K, Fischer PU, Hoerauf A, et al. The long and winding road towards new treatments against lymphatic filariasis and onchocerciasis. Trends Parasitol. 2024;40:829–45. 10.1016/j.pt.2024.07.005. 39122645 10.1016/j.pt.2024.07.005

[8] Bandi C, Anderson TJ, Genchi C, Blaxter ML. Phylogeny of *Wolbachia* in filarial nematodes. Proc Biol Sci. 1998;265:2407–13. 10.1098/rspb.1998.0591. 9921679 10.1098/rspb.1998.0591 PMC1689538

[9] Henkle-Dührsen K, Eckelt VH, Wildenburg G, Blaxter M, Walter RD. Gene structure, activity and localization of a catalase from intracellular bacteria in *Onchocerca volvulus*. Mol Biochem Parasitol. 1998;96:69–81. 10.1016/s0166-6851(98)00109-1. 9851608 10.1016/s0166-6851(98)00109-1

[10] Lefoulon E, Bain O, Makepeace BL, d’HaeseUni CS, Martin C, Gavotte L. Breakdown of coevolution between symbiotic bacteria *Wolbachia* and their filarial hosts. PeerJ. 2016;4:e1840. 10.7717/peerj.1840. 27069790 10.7717/peerj.1840 PMC4824920

[11] Bandi C, McCall JW, Genchi C, Corona S, Venco L, Sacchi L. Effects of tetracycline on the filarial worms *Brugia pahangi* and *Dirofilaria immitis* and their bacterial endosymbionts *Wolbachia*. Int J Parasitol. 1999;29:357–64. 10.1016/s0020-7519(98)00200-8. 10221636 10.1016/s0020-7519(98)00200-8

[12] Hoerauf A, Nissen-Pähle K, Schmetz C, Henkle-Dührsen K, Blaxter ML, Büttner DW, et al. Tetracycline therapy targets intracellular bacteria in the filarial nematode *Litomosoides sigmodontis* and results in filarial infertility. J Clin Invest. 1999;103:11–8. 10.1172/JCI4768. 9884329 10.1172/JCI4768 PMC407866

[13] Langworthy NG, Renz A, Mackenstedt U, Henkle-Dührsen K, de Bronsvoort MB, Tanya VN, et al. Macrofilaricidal activity of tetracycline against the filarial nematode *Onchocerca ochengi*: elimination of *Wolbachia* precedes worm death and suggests a dependent relationship. Proc Biol Sci. 2000;267:1063–9. 10.1098/rspb.2000.1110. 10.1098/rspb.2000.1110 PMC1690645 10885510

[14] Townson S, Hutton D, Siemienska J, Hollick L, Scanlon T, Tagboto SK, et al. Antibiotics and *Wolbachia* in filarial nematodes: antifilarial activity of rifampicin, oxytetracycline and chloramphenicol against *Onchocerca gutturosa, Onchocerca lienalis* and *Brugia pahangi*. Ann Trop Med Parasitol. 2000;94:801–16. 10.1080/00034980020027988. 11214099 10.1080/00034980020027988

[15] Anti-Wolbachia Consortium: A-WOL. https://a-wol.net.

[16] Kramer LH, Passeri B, Corona S, Simoncini L, Casiraghi M. Immunohistochemical/immunogold detection and distribution of the endosymbiont *Wolbachia* of *Dirofilaria immitis* and *Brugia pahangi* using a polyclonal antiserum raised against WSP (*Wolbachia* surface protein). Parasitol Res. 2003;89:381–6. 10.1007/s00436-002-0765-6. 12632152 10.1007/s00436-002-0765-6

[17] Kramer LH, Tamarozzi F, Morchón R, López-Belmonte J, Marcos-Atxutegi C, Martín-Pacho R, et al. Immune response to and tissue localization of the *Wolbachia* surface protein (WSP) in dogs with natural heartworm (*Dirofilaria immitis*) infection. Vet Immunol Immunopathol. 2005;106:303–8. 10.1016/j.vetimm.2005.03.011. 15876457 10.1016/j.vetimm.2005.03.011

[18] Bazzocchi C, Ceciliani F, McCall JW, Ricci I, Genchi C, Bandi C. Antigenic role of the endosymbionts of filarial nematodes: IgG response against the *Wolbachia* surface protein in cats infected with *Dirofilaria immitis*. Proc Biol Sci. 2000;267:2511–6. 10.1098/rspb.2000.1313. 11197127 10.1098/rspb.2000.1313 PMC1690852

[19] Kramer L, Simón F, Tamarozzi F, Genchi M, Bazzocchi C. Is *Wolbachia* complicating the pathological effects of *Dirofilaria immitis* infections? Vet Parasitol. 2005;133:133–6. 10.1016/j.vetpar.2005.04.011. 15885912 10.1016/j.vetpar.2005.04.011

[20] Turner JD, Langley RS, Johnston KL, Gentil K, Ford L, Wu B, et al. *Wolbachia *lipoprotein stimulates innate and adaptive immunity through Toll-like receptors 2 and 6 to induce disease manifestations of filariasis. J Biol Chem. 2009;284:22364–78. 10.1074/jbc.M901528200. 19458089 10.1074/jbc.M901528200 PMC2755959

[21] Gillette-Ferguson I, Daehnel K, Hise AG, Sun Y, Carlson E, Diaconu E, et al. Toll-like receptor 2 regulates CXC chemokine production and neutrophil recruitment to the cornea in *Onchocerca volvulus*/*Wolbachia*-induced keratitis. Infect Immun. 2007;75:5908–15. 10.1128/IAI.00991-07. 17875630 10.1128/IAI.00991-07 PMC2168349

[22] Gillette-Ferguson I, Hise AG, McGarry HF, Turner J, Esposito A, Sun Y, et al. *Wolbachia*-induced neutrophil activation in a mouse model of ocular onchocerciasis (river blindness). Infect Immun. 2004;72:5687–92. 10.1128/IAI.72.10.5687-5692.2004. 15385467 10.1128/IAI.72.10.5687-5692.2004 PMC517527

[23] Daehnel K, Gillette-Ferguson I, Hise AG, Diaconu E, Harling MJ, Heinzel FP, et al. Filaria/*Wolbachia* activation of dendritic cells and development of Th1-associated responses is dependent on Toll-like receptor 2 in a mouse model of ocular onchocerciasis (river blindness). Parasite Immunol. 2007;29:455–65. 10.1111/j.1365-3024.2007.00962.x. 17727569 10.1111/j.1365-3024.2007.00962.x

[24] Tamarozzi F, Wright HL, Johnston KL, Edwards SW, Turner JD, Taylor MJ. Human filarial *Wolbachia* lipopeptide directly activates human neutrophils in vitro. Parasite Immunol. 2014;36:494–502. 10.1111/pim.12122. 24909063 10.1111/pim.12122 PMC4282327

[25] Tamarozzi F, Turner JD, Pionnier N, Midgley A, Guimaraes AF, Johnston KL, et al. *Wolbachia* endosymbionts induce neutrophil extracellular trap formation in human onchocerciasis. Sci Rep. 2016;6:35559. 10.1038/srep35559. 27752109 10.1038/srep35559 PMC5067710

[26] Bazzocchi C, Mortarino M, Grandi G, Kramer LH, Genchi C, Bandi C, et al. Combined ivermectin and doxycycline treatment has microfilaricidal and adulticidal activity against *Dirofilaria immitis* in experimentally infected dogs. Int J Parasitol. 2008;38:140–210. 10.1016/j.ijpara.2008.03.002. 10.1016/j.ijpara.2008.03.002 18433753

[27] Kramer L, Grandi G, Leoni M, Passeri B, McCall J, Genchi C, et al. *Wolbachia* and its influence on the pathology and immunology of *Dirofilaria immitis* infection. Vet Parasitol. 2008;158:191–5. 10.1016/j.vetpar.2008.09.014. 18947926 10.1016/j.vetpar.2008.09.014

[28] Boreham PF. Activation in vitro of some biological systems by extracts of adult worms and microfilariae of *Dirofilaria immitis*. J Helminthol. 1984;58:207–12. 10.1017/s0022149x00026985. 6542116 10.1017/s0022149x00026985

[29] American Heartworm Society Canine Guidelines for the Prevention, Diagnosis, and Management of Heartworm (*Dirofilaria immitis*) Infection in Dogs. 2025. https://www.heartwormsociety.org/resources/54-heartworm-guidelines/375-canine-heartworm-guidelines. Accessed 23 Oct 2025.

[30] European Society of Dirofilariasis and Angiostrongylosis Guidelines for Clinical Management of Canine Heartworm Disease. 2017. https://www.esda.vet/guidelines.html. Accessed 23 Oct 2025.

[31] Nelson CT, Myrick ES, Nelson TA. Clinical benefits of incorporating doxycycline into a canine heartworm treatment protocol. Parasit Vectors. 2017;10:515. 10.1186/s13071-017-2446-4. 29143657 10.1186/s13071-017-2446-4 PMC5688473

[32] Dixon-Jimenez AC, Coleman AE, Rapoport GS, Creevy KE, Roth I, Correa M, et al. Approaches to canine heartworm disease treatment among alumni of a single college of veterinary medicine. J Am Anim Hosp Assoc. 2018;54:246–56. 10.5326/JAAHA-MS-6601. 30040440 10.5326/JAAHA-MS-6601

[33] Jacobson LS, DiGangi BA. An accessible alternative to melarsomine: “moxi-doxy” for treatment of adult heartworm infection in dogs. Front Vet Sci. 2021;8:702018. 10.3389/fvets.2021.702018. 34386540 10.3389/fvets.2021.702018 PMC8353148

[34] Ames MK, VanVranken P, Evans C, Atkins CE. Non-arsenical heartworm adulticidal therapy using topical moxidectin-imidacloprid and doxycycline: a prospective case series. Vet Parasitol. 2020;282:109099. 10.1016/j.vetpar.2020.109099. 32450463 10.1016/j.vetpar.2020.109099

[35] WHO. Progress in eliminating onchocerciasis in the WHO Region of the Americas: doxycycline treatment as an end-game strategy. Weekly Epidemiol Rec. 2021;94:415–9.

[36] Johnston KL, Hong WD, Turner JD, O’Neill PM, Ward SA, Taylor MJ. Anti-*Wolbachia* drugs for filariasis. Trends Parasitol. 2022;37:1068–81. 10.1016/j.pt.2021.06.004. 10.1016/j.pt.2021.06.004 34229954

[37] Godel C, Kumar S, Koutsovoulos G, Ludin P, Nilsson D, Comandatore F, et al. The genome of the heartworm, *Dirofilaria immitis*, reveals drug and vaccine targets. FASEB J. 2012;26:4650–61. 10.1096/fj.12-205096. 22889830 10.1096/fj.12-205096 PMC3475251

[38] Hong WD, Benayoud F, Nixon GL, Ford L, Johnston KL, Clare RH, et al. AWZ1066S, a highly specific anti-*Wolbachia* drug candidate for a short-course treatment of filariasis. Proc Natl Acad Sci U S A. 2019;116:1414–9. 10.1073/pnas.1816585116. 30617067 10.1073/pnas.1816585116 PMC6347715

[39] Marriott AE, Dagley JL, Hegde S, Steven A, Fricks C, DiCosty U, et al. Dirofilariasis mouse models for heartworm preclinical research. Front Microbiol. 2023;14:1208301. 10.3389/fmicb.2023.1208301. 37426014 10.3389/fmicb.2023.1208301 PMC10324412

[40] Grandi G, Morchon R, Kramer L, Kartashev V, Simon F. *Wolbachia* in *Dirofilaria repens*, an agent causing human subcutaneous dirofilariasis. J Parasitol. 2008;94:1421–3. 10.1645/GE-1575.1. 19127968 10.1645/GE-1575.1

[41] Giannelli A, Ramos RA, Traversa D, Brianti E, Annoscia G, Bastelli F, et al. Treatment of *Dirofilaria repens* microfilariaemia with a combination of doxycycline hyclate and ivermectin. Vet Parasitol. 2013;197:702–4. 10.1016/j.vetpar.2013.05.012. 23768566 10.1016/j.vetpar.2013.05.012

[42] Lechner AM, Gastager H, Kern JM, Wagner B, Tappe D. Case report: successful treatment of a patient with microfilaremic dirofilariasis using doxycycline. Am J Trop Med Hyg. 2020;102:844–6. 10.4269/ajtmh.19-0744. 32043447 10.4269/ajtmh.19-0744 PMC7124902

[43] Vranjković MP, Vitko Havliček A, Kramar M, Balen Topić M, Beck D, Jurković Žilić D, et al. Not a dead-end host: first confirmed persistent microfilaremia in human *Dirofilaria repens* infection. Microorganisms. 2025;13:2263. 10.3390/microorganisms13102263. 41156723 10.3390/microorganisms13102263 PMC12565814

[44] American Heartworm Society. Current feline guidelines for the prevention, diagnosis, and management of heartworm (*Dirofilaria immitis*) infection in cats. Holly Springs, NC: American Heartworm Society; 2024.

[45] Keiser PB, Coulibaly Y, Kubofcik J, Diallo AA, Klion AD, Traoré SF, et al. Molecular identification of *Wolbachia* from the filarial nematode *Mansonella perstans*. Mol Biochem Parasitol. 2008;160:123–8. 10.1016/j.molbiopara.2008.04.012. 18538871 10.1016/j.molbiopara.2008.04.012 PMC2497444

